# Human Activity Recognition in the Presence of Occlusion

**DOI:** 10.3390/s23104899

**Published:** 2023-05-19

**Authors:** Ioannis Vernikos, Theodoros Spyropoulos, Evaggelos Spyrou, Phivos Mylonas

**Affiliations:** 1 Department of Informatics and Telecommunications, University of Thessaly, 35131 Lamia, Greece; 2 Department of Digital Systems, University of Piraeus, 18534 Piraeus, Greece; 3 Department of Informatics and Computer Engineering, University of West Attica, Egaleo Park, 12243 Athens, Greece

**Keywords:** human activity recognition, occlusion, deep learning, convolutional neural networks

## Abstract

The presence of occlusion in human activity recognition (HAR) tasks hinders the performance of recognition algorithms, as it is responsible for the loss of crucial motion data. Although it is intuitive that it may occur in almost any real-life environment, it is often underestimated in most research works, which tend to rely on datasets that have been collected under ideal conditions, i.e., without any occlusion. In this work, we present an approach that aimed to deal with occlusion in an HAR task. We relied on previous work on HAR and artificially created occluded data samples, assuming that occlusion may prevent the recognition of one or two body parts. The HAR approach we used is based on a Convolutional Neural Network (CNN) that has been trained using 2D representations of 3D skeletal motion. We considered cases in which the network was trained with and without occluded samples and evaluated our approach in single-view, cross-view, and cross-subject cases and using two large scale human motion datasets. Our experimental results indicate that the proposed training strategy is able to provide a significant boost of performance in the presence of occlusion.

## 1. Introduction

Human activity recognition (HAR) from visual data is considered a very challenging computer vision task [1], while it has been continuously attracting increasing attention within the research community [2]. It typically aims to recognize human motion or behavior within a video sequence, using data captured by a camera. In the following, we will define an “activity” as motion performed by a human, taking place within a relatively short (however, not instant) time period and involving multiple body parts [2]. The aforementioned informal definition may be expanded to also include interactions between a human and an object or between two or more humans (group activities). Additionally, note that it does not include gestures, as they require a relatively small (“instant”) amount of time, while typically requiring a single body part.

There exist several HAR applications. The most notable ones include content-based video summarization (e.g., for retrieval of activities), human–computer interaction (e.g., for interaction based on users’ pose and/or motion), education (e.g., for monitoring students’ attendance), healthcare (e.g., for fall detection), video surveillance (e.g., for abnormal activity recognition), and sports (e.g., for identifying players’ actions) [3]. We should note that several HAR applications require the use of either wearable sensors or environmental sensors (i.e., installed in the subject’s environment). Typical wearable sensors include smartwatches, hand/body worn sensors, smartphones, etc. Additionally, typical environmental sensors include video/thermal cameras, microphones, infrared, pressure, magnetic, and RFID sensors [4].

Although wearable sensors may combine adequate performance with low cost, they are not preferred by users and their usability is below average [5,6]. Moreover, it is common sense that, e.g., in the case of a home environment, overloading it with a large number of sensors may be both expensive and unwanted by the users, since it would require many interventions in home furniture or appliances. Thus, a common approach to combining low cost with minimal interventions is to use only cameras, whose role is to capture users’ motion. However, the undesired trade-off is that, although their performance may be excellent in laboratory conditions, in real-life environments they exhibit a performance loss, as they suffer from viewpoint and illumination changes and occlusion.

The problem of viewpoint changes and their effect on HAR has been extensively studied in our previous work [7]. Specifically, we dealt with the problem of viewpoint invariance and demonstrated that the decrease of accuracy due to viewpoint changes may be limited when using more than one camera, allowing for satisfactory results in real-life applications. Moreover, due to recent advances in technology and specifically in camera sensors, recent cameras perform significantly better in low-light conditions compared to previous models. Additionally, some cameras (often called “RGB+depth” cameras), complementary to color video, also provide depth information, resulting in robust extraction of human figures, e.g., as a set of 3D points, and even in poor illumination conditions. Therefore, from the three aforementioned problems, occlusion is the one that introduces the most limitations.

To understand the notion of occlusion, let us consider two objects in a 3D space. Given a certain viewpoint, occlusion occurs when one of the objects partially/fully blocks the view of the second. In the context of HAR, occlusion may occur, e.g., in cases when the subject performs an activity and is occluded by a physical object such as furniture or by another person. Regarding its effect on HAR, let us consider the (extreme) case of a subject performing the activity “handwaving,” while the involved arm and hand are not visible. Clearly, in order to recognize this activity, crucial information regarding the subject’s motion is missing. This case might be excessive, but there are applications such as, e.g., surveillance systems in crowded places such as metro stations where an activity of a subject might not be recognized due to the occlusion of some of their body parts by the crowd surrounding her/him. Occlusion may also be temporally partial, i.e., one or more body parts may be occluded but not during the whole activity. This may happen, e.g., in the case of a moving subject.

In this work, our goal was to assess how partial occlusion of the subject affects the accuracy of HAR and to overcome its effect. To the best of our knowledge, public motion-based datasets that may be used for HAR [8,9] have been created under ideal laboratory conditions, which include the prevention of any type of occlusion. Thus, since the creation of a large scale dataset is a time consuming task, we decided to follow an approach such as that of Gu et al. [10]. More specifically, we simulated occlusion by manually discarding subsets of joints that correspond to one/two body parts of the subject, while she/he is performing a given activity. We assumed that these parts remain occluded during the whole activity, that is, we did not consider the case of temporally partial occlusion. The contribution of this work is twofold: (a) we extended the small-scale evaluation of our previous work [11], where we assessed how partial occlusion of the subject affects the accuracy of HAR focusing on Activities of Daily Living (ADLs) [12]; herein, we evaluated it using two challenging large-scale human motion datasets [8,9]; (b) we tried to overcome the effect of occlusion by incorporating occluded samples in our training procedure.

The rest of this paper is organized as follows: in Section 2, we present research works that aimed to assess or even tackle the effect of occlusion in HAR-related scenarios. Then, in Section 3, we present the proposed methodology for simulating occlusion, for assessing its effect on HAR and for overcoming its effect. Experimental results are presented and discussed in Section 4, while conclusions are drawn in Section 5, wherein plans for future work are also presented.

## 2. Related Works

As has already been mentioned, the problem of HAR is one of the most challenging research areas in computer vision; therefore, a large amount of research has been conducted on this subject during recent years. The majority of them have been based on 2D representations of skeletal motion which are then used as input to CNNs [13,14,15,16,17,18]. An extensive survey was conducted by Wang et al. [2]. Interestingly, although it is widely accepted, as it is intuitive, that occlusion constitutes one of the most important factors that compromise the performance of HAR approaches [19], rendering them to produce poor and in extreme cases unusable results, there do not exist many studies that focus on studying its effects on the performance of HAR approaches or even attempt to propose methods so as to overcome them.

In previous work [11], we have extensively studied the effect of occlusion in the task of HAR. We relied on the HAR approach of Papadakis et al. [20] and simulated occlusion upon removing one or more body parts from 3D human skeletons and during the whole activity. Throughout our experiments, we used a Convolutional Neural Network that had been trained without using any occluded samples. Specifically, we considered the cases of removing one or both arms, one or both legs, and also the cases of removing the arm and the leg of the same side. Our experimental evaluation proved that the removal of one or both arms led to a drop of performance, which in some cases was considered quite severe. However, we concluded that this was not a surprise, as most of the activities considered therein were expressed mainly by one or both arms’ motion. In the following, we shall attempt to present the approaches that have dealt with occlusion and their main findings.

Angelini et al. [21] proposed an HAR approach that relied on OpenPose [22] for pose extraction. Specifically, they extracted both low- and high-level features from body poses using a 1D CNN and an LSTM for classification. They used real CCTV data where part of the body was occluded; however, they also created synthetic occluded data by removing body parts. They experimented by incorporating occluded samples in training and showed that, in most cases, this strategy is able to provide a boost of performance in cases of both occlusion and missing data. They experimented on multiple datasets, i.e., i3DPost [23], IXMAS [24], KTH [25] etc., while they also created their own dataset called ISLD, comprising classes such as bend, box, hand-clap, one-hand wave, run, walk, etc.

Iosifidis et al. [19] used a multi-camera setup, surrounding the subject from all sides. They trained a model using data from all available cameras and then, in order to simulate occlusion, they used a subset of those cameras. Their main idea was that, due to occlusion, not all cameras are able to simultaneously capture the subject’s motion, yet in all cases more than one camera manages to capture the whole body of the subject. In addition, the recognition of an activity was performed using the combined results of cameras that had not been “affected” by any occlusion, while those that had been omitted “suffered” from occlusion. They conducted experiments using two publicly available datasets, the i3DPost [23], comprising eight daily activities, and the AIIA-MOBISERV, [26] comprising three meal activities. The proposed method has been evaluated for both single-view and multi-view action classification problems, providing results with sufficient accuracy.

Li et al. [27] used manually defined occlusion areas and proposed a new encoding technique to transform a skeleton into a feature matrix and integrated an attention model into a Generative Adversarial Network, focusing on completing the missing data of the feature matrix, rather than the exact skeleton. For classification, they used a Residual Network (ResNet). To evaluate their approach, they used a custom dataset of eight actions of construction workers, such as driving a truck, transporting cement, paving concrete, etc. Additionally, Li et al. [28] proposed an approach based on action graphs so as to capture the dynamics of actions and used a bag-of-3D points so as to describe the salient postures that correspond to the nodes of the action graph. They simulated occlusion by first dividing the depth map into four quadrants and then by ignoring 3D points falling into the specified quadrants that are considered to be affected by occlusion. They did not include any occluded samples in training. They used a dataset comprising 20 actions such as high arm wave, horizontal arm wave, hammer, etc. Their results showed that, unless the critical quadrants were omitted, the performance drop was relatively small.

Similarly, Gu et al. [10] generated occlusion masks which were used both in training and evaluation, so as to simulate occlusion in more than one 2D skeletal joint. Moreover, they attempted to reconstruct the missing skeleton parts using a regression network. They evaluated their work using their own dataset, namely MMHuman, comprising six classes (e.g., Kung Fu, shake hand, etc.). They also demonstrated that their approach is able to outperform previous pose estimation methods on the Human 3.6 M dataset [29]. Finally, Yang et al. [30] proposed to pre-train a single pose embedding network, namely OR-VPE, whose role was to learn occlusion-robust representations for pose sequences. They simulated occlusions using an augmentation mechanism whose goal was to randomly prune the spatio-temporal skeleton structure. They used an encoder in order to create the pose embeddings in a latent space and a contrastive module to render this space occlusion-invariant. They evaluated their approach using the NTU-RGB+D dataset [9] and also the Toyota Smarthome [31], N-UCLA [32], and Penn Action [33] datasets.

## 3. Proposed Methodology

### 3.1. Visual Data

**Figure 1:**
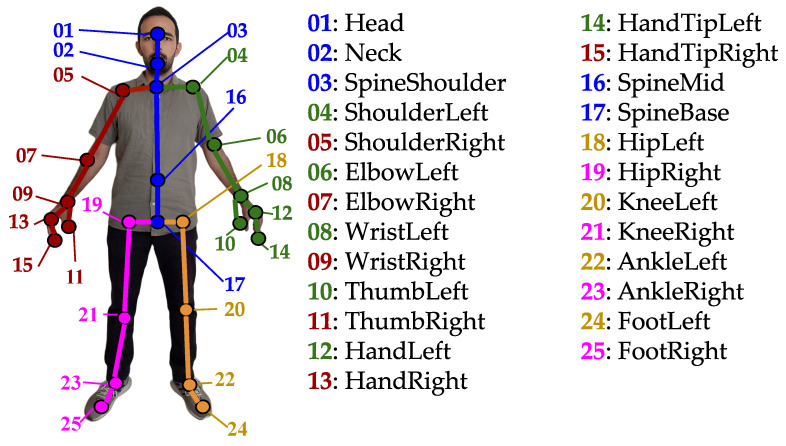
The 25 skeletal joints extracted by Microsoft Kinect v2. The joints and edges that correspond to the 5 main body parts, namely head-torso, right arm, left arm, right leg, and left leg are colored in blue, red, green, orange, and purple, respectively.

**Figure 2:**
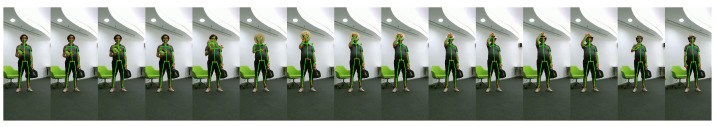
A sequence of an actor performing the activity *wearing a hat*. Extracted human skeleton 3D joints using the Kinect SDK have been overlaid. Frames have been taken from the NTU dataset [9] and have been trimmed for illustration purposes.

**Figure 3:**
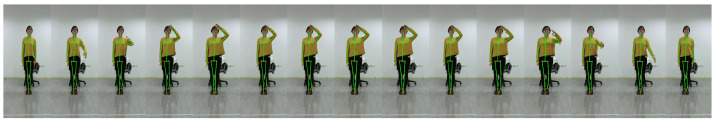
A sequence of an actor performing the activity *salute*. Extracted human skeleton 3D joints using the Kinect SDK have been overlaid. Frames have been taken from the PKU-MMD dataset [8] and have been trimmed for illustration purposes.

In this work, we considered 3D skeleton data that were captured by the Microsoft Kinect v2 camera. Specifically, these data consist of the 3D spatial coordinates of a set of 25 human skeleton joints that were captured during the recording of an action over time, for each subject in the scene and for a maximum of six subjects. The human skeleton comprises these joints and was modelled as a graph; the joints are its nodes, corresponding to parts such as head, shoulders, knees etc. and are connected by edges that follow the body structure. An illustration of the human skeleton joints and edges that were extracted by Kinect v2 are illustrated in Figure 1. Note that, following the example of [10,11], we divided the skeleton into its five main body parts, namely head-torso, right arm, left arm, right leg, and left leg, since one or more will be removed as an effect of occlusion. Therefore, in Figure 1 we illustrate the skeleton, highlighting these parts. In Figure 2 and Figure 3, we illustrate the RGB and the skeletal data of two sequences of actors performing activities.

### 3.2. Image Representation of Skeletal Data

Since our goal was to use a CNN for the classification of activities, a mandatory step was to represent the 3D skeletal motion with a 2D image, so that the latter would capture and preserve the spatio-temporal properties of this motion. Of course, such representations should be able to discriminate among different activities, while also complying with the graph structure of the skeleton representation that is used. Therefore, and based on our previous work [34], we adopted a pseudo-colored representation of the skeletal motion that aimed to capture the varying inter-joint distances during the performance of an activity.

**Figure 4:**
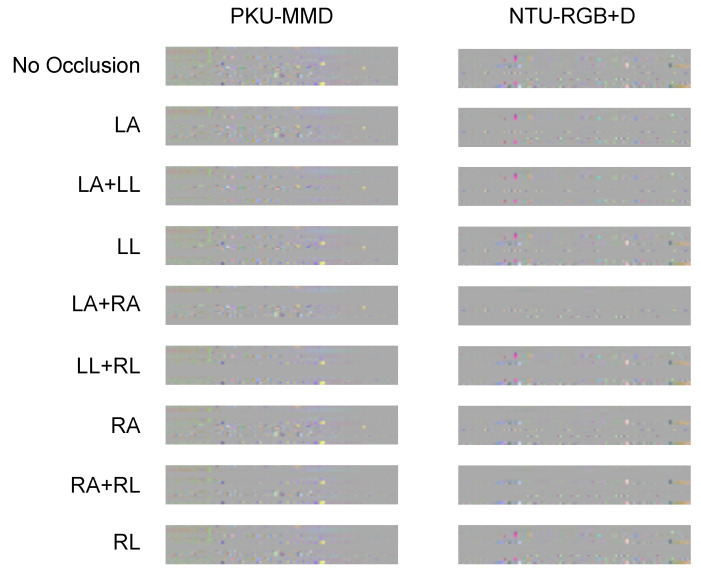
Pseudo-coloured images for the PKU-MMD [8] and the NTU [9] datasets. LA, RA, LL, and RL denote the cases of occlusion of left arm, right arm, left leg, and right leg.

Specifically, let x(n), y(n) and z(n), n=1,…,N denote the sequences of the x,y,z co-ordinates of the skeletal joint motion at the *n*-th frame and occurring within a period of time of *N* frames. Since Kinect v2 extracts 25 joints, this motion “produces” a set of 75 such sequences. Prior to the creation of the image representation, there is the need to address the problem of temporal variability. It should be intuitive that (a) different activities require different amounts of time and (b) the same activity requires different amounts of time both in the cases it is performed by different subjects and even when it is performed by the same subject. As in [20], we used a linear interpolation step, setting the number of frames *F* as equal for all activity examples. Upon performing several experiments, we set F=150. In order to create the pseudo-colored images, spatial co-ordinates *x*, *y*, *z* were assigned to R, G, B color channels of the resulting pseudo-colored image, respectively. The procedure of creating these images was as follows: let *R* denote the red channel, *i* the *i*-th frame, and j the *j*-th joint. The equation used to create the *R*-value of pixel (i,j) is: R(i,j)=R(i+1,j)−R(i,j). Similarly, *G* and *B* values were created. In Figure 4, examples of these pseudo-colored images are illustrated.

### 3.3. Occlusion of Skeletal Data

As we have already discussed, in real-life scenarios, occlusion may be one of the most crucial causes of the poor performance of HAR approaches. Prior to describing our approach to simulating occlusion, we first discuss the main causes. Let us consider an assistive living scenario, where the subject’s behavior is monitored by cameras. In many occasions, actions take place behind furniture or more than one person is present in the same room. Thus, it is expected that occlusion is often occurring, incommoding the task of recognition algorithms, as it is the main factor for the loss of visual information. Even in cases of simple activities that involve one or two body parts, it is obvious that by occluding them, i.e., by losing the information regarding their motion, recognition algorithms are prone to failure.

As we have already mentioned, the goal of this work was to assess whether the performance drop caused by occlusion may be limited upon adding occluded samples to the training process. First, we should emphasize that most publicly available, large scale datasets such as the ones we used for the evaluation of this work, i.e., NTU RGB+D [9] and the PKU-MMD [8], have been created in laboratory (i.e., ideal) conditions. Thus, there exist no data samples that are affected by occlusion, while room illumination is appropriate for video capturing. We assumed that these datasets are “clean,” i.e., they do not contain any samples with missing/noisy data and the only limitations imposed are due to the capabilities of Kinect v2, which was used for data collection.

In order to simulate the effect of occlusion, i.e., to create artificially occluded activity samples, we followed the paradigms of [10,11] and we discarded subsets of the skeleton that correspond to meaningful body parts. Specifically, as we have already discussed in Section 3.1, we considered arms (each comprising shoulder, elbow, wrist, hand, and hand-tip), legs (each comprising hip, knee, ankle, and foot), and the torso (comprising head, neck, spine-shoulder, spine-mid, and spine-base). This way, we formed five body parts, as illustrated in Figure 1. We should herein note that, in our case, we assumed that occlusion affects the whole activity, i.e., the body parts that were occluded remained occluded during the whole activity duration.

**Figure 5:**
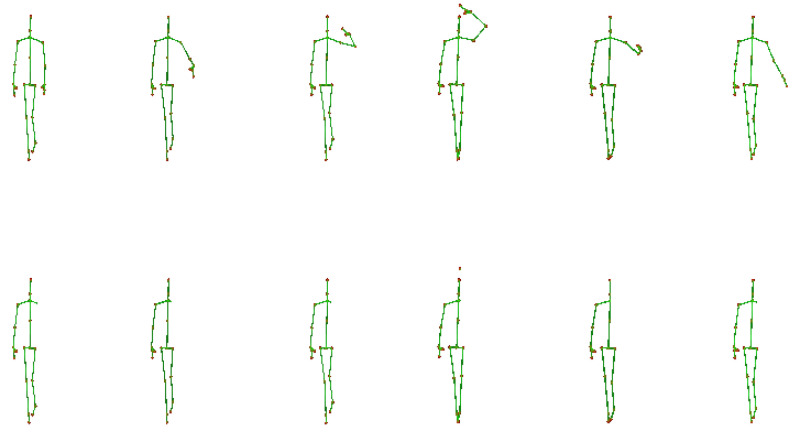
Example skeleton sequences of the activity *salute* from the PKU-MMD dataset [8]. First row: skeletons include all 25 joints; second row: joints corresponding to the left arm have been discarded.

**Figure 6:**
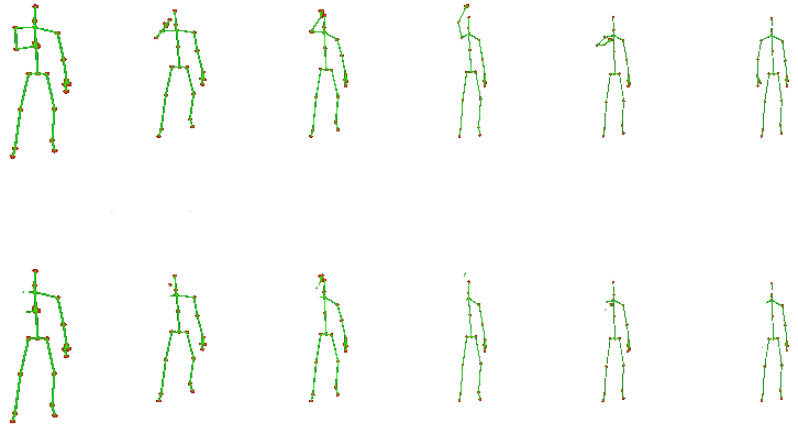
Example skeleton sequences of the activity *wearing a hat* from the NTU dataset [9].First row: skeletons include all 25 joints; second row: joints corresponding to the left arm have been discarded.

We considered the cases of occlusion of: (a) an arm; (b) a leg; (c) both arms; (d) both legs; and (e) an arm and a leg. Intuitively, when two parts are occluded, the most expected case is that they both are from the same side. Moreover, our initial experiments indicated that by removing the torso the accuracy was not significantly affected. Therefore, throughout our experiments, the torso was always present. An example of an activity with and without the occlusion of a body part is illustrated in Figure 5 and Figure 6. In this example, it is evident that a single body part may carry significant information regarding the activity.

Note that several research works among those that have been presented in Section 2 also used occluded samples within the training process [10,21,35]. However, in [21], only random skeletal joints were removed; yet these did not correspond to structured body parts as in our case. Moreover, in [10], occlusion was not continuous as in our case; instead it may go from partial to full during an activity, yet within several temporal periods, no occlusion is present. Additionally, contrary to [19], where due to occlusion only some cameras were affected, in our approach, all available cameras were similarly affected.

### 3.4. Classification

**Figure 7:**
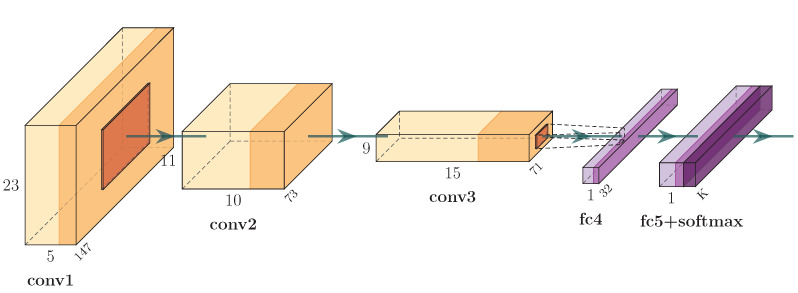
The Convolutional Neural Network that has been used in this work.

For classification, we used a Convolutional Neural Network (CNN). Specifically, the architecture of the CNN that has been used throughout our experiments has been experimentally defined and was initially used in previous works [20,34]. It consists of a 2D convolutional layer that filters the 25×150 input image with five kernels of 3×3 size, a max-pooling layer that performs 2×2 subsampling, two consecutive convolutional layers of size 3×3 with 10 and 15 kernels, a max-pooling layer performing 2×2 subsampling, a flattened layer that transforms the output of the last pooling layer into a vector, which consists of the input to a dense layer upon applying a dropout layer with a dropout rate equal to 0.5 and a second dense layer producing the output of the network. The CNN is illustrated in Figure 7.

## 4. Experiments and Results

In this section, we present and discuss the results of the experimental evaluation of the proposed approach, using two publicly available and well-known human activity recognition datasets. The goal of this evaluation was to assess whether the inclusion of occluded samples within the training process may improve the performance of classification strategies. We relied on our previous work [34], where we presented an image representation of 3D skeletal motion. Moreover, we used the same CNN architecture for classification as in [36]. Note that the goal of the following experiments was not to provide state-of-the-art results in human activity recognition, but to be used as a baseline so as to verify the effectiveness of our approach.

### 4.1. Datasets

For the experimental evaluation of our work, we used two widely known and freely available datasets. More specifically, we used:

a. PKU-MMD [8], which is an action recognition dataset that contains 1076 long video sequences in 51 action categories, performed by 66 subjects and in three camera views. The total amount of the action instances is approximately 20,000. For the recording of the dataset they used the Kinect v2 sensor. The 51 action categories are divided into two parts: 41 daily actions (e.g., brushing hair, brushing teeth, eating a meal, etc.) and 10 interaction actions (e.g., handshaking, hugging another person, slapping another person, etc.). For the data collection, three cameras with different viewpoints ($-45^{\circ }$, $0^{\circ }$, $+45^{\circ }$) were used.
b. NTU-RGB+D [9], which is a large-scale RGB+D action recognition dataset containing approx. 57,000 video samples from 60 action classes and from 40 distinct subjects. The dataset contains 40 daily actions (drop, stand up, play with phone, etc.), 11 mutual actions (punch, pushing, hugging, etc.), and nine health-related actions (sneeze, nausea, neck pain, etc.). For data collection, the same camera setup as in PKU-MMD was used.

### 4.2. Experimental Setup and Implementation Details

The experiments were performed on a workstation with an Intel core™i7 5820K 12-core processor on 3.3 GHz and 16 GB RAM, using NVIDIA™GeForce RTX 2060 SUPER GPU with 8 GB and Ubuntu 20.04 (64 bit). All pipelines were implemented in Python, using Keras 2.6 [37] and Tensorflow 2.3 [38] backend. The batch size used consisted of 32 and 80 epochs. The optimizer used was the RMSprop, while the categorical cross-entropy loss was adopted. The data split for training, validation, and testing followed exactly the one proposed by the datasets’ authors [8,9].

### 4.3. Evaluation Protocol and Results

For the evaluation of the proposed approach, we studied eight cases of missing body parts of the skeleton, as we have discussed in Section 3.3. Specifically, we performed three types of experiments for comparison purposes:

a. Training and test sets consisting of full body parts only;
b. Training set consisting of full body parts only, test set consisting of occluded body parts only;
c. Training set consisting of full and occluded body parts, test set consisting of occluded body parts.

Following typical evaluation protocols encountered in multi-camera datasets as those used herein [8,9], we further split the aforementioned experiments into three types:

a. Per camera position (single view), where training and test sets derive from the same camera (viewpoint), e.g., both deriving from the middle camera;
b. Cross view experiments, where training and test sets derive from different cameras (viewpoints), e.g., training deriving from the middle camera, testing deriving from the left camera;
c. Cross subject experiments, wherein subjects are split into training and testing groups, i.e., each subject appears in exactly one of these groups.

We performed experiments using the full PKU-MMD [8] and NTU-RGB+D [9] datasets. We also performed experiments using subsets of both these datasets, as follows:

a. From the PKU-MMD dataset we selected 11 actions related to activities of daily living: eating a meal/snack, falling, handshaking, hugging another person, making a phone call/answering the phone, playing with phone/tablet, reading, sitting down, standing up, typing on a keyboard, and wearing a jacket;
b. From the NTU-RGB+D dataset we selected 12 medical conditions: sneezing/coughing, staggering, falling down, headache, chest pain, back pain, neck pain, nausea/vomiting, fanning self, yawning, stretching oneself, and blowing the nose.

In all cases, we measured F${}_{1}$ score and accuracy. Results of the proposed methodology are depicted in Table 1 for the 11 activities of daily living of the PKU-MMD dataset, in Table 2 for the 12 medical conditions of NTU-RGB+D dataset, and in Table 3 and Table 4 for the full PKU-MMD and NTU-RGB+D datasets, respectively.

Starting with the PKU-MMD dataset and based on the results depicted in Table 1 and Table 3, we observe the following:

- In almost all experiments, both the F${}_{1}$ score and the accuracy of the proposed approach are improved compared to the “simple” case of occlusion;
- In general, it is also comparable to the baseline case;
- The only exception to the above-mentioned are the cases of cross-view experiments when using:
  − Only the 11 activities of daily living, and specifically in the case where the left and the middle cameras were used for training, while the right one was used for testing; and
  − The full PKU-MMD dataset, and specifically in the case where the left camera was used for training, while the right one was used for testing;
- As expected, and due to the activities involved, in cases of the occlusion of legs, a drop of performance is not observed, in general.

In the case of the NTU-RGB+D dataset and based on the results depicted in Table 2 and Table 4, we observe the following:

- In cases where both metrics indicated improved performance, compared to the “simple” case of occlusion:
- In general, in that case it is also comparable to the baseline case;
- The only exception to the above-mentioned are the cases of the cross-view experiments when using:
  − Only the 12 medical conditions, and specifically in the case where the middle camera was used for training, while the right one was used for testing or vice versa;
  − The full NTU-RGB+D dataset, and specifically in the case of single view;
  − A few combinations of training and testing in the cross-view case;
- Additionally, in this case and as expected, in the case of the occlusion of legs, a drop of performance is not observed, in general.

A notable observation of our experimental results indicates that, due to data augmentation that is indirectly caused by the addition of the occluded samples in the training phase, in many cases and in both datasets we may observe that the results of the proposed approach, i.e., when the training set is augmented with occluded samples, typically lead to higher accuracy and F${}_{1}$ score. However, this result was not anticipated prior to the experimental evaluation. Of course, since it is well-known that data augmentation may typically boost the performance of deep architectures, this result is adequately explained this way.

## 5. Conclusions and Future Work

In this paper, we dealt with the problem of human activity recognition in the presence of occlusion, which still remains an aspect that has not yet received significant attention, while it compromises performance. Our goal was twofold: (a) we intended to extensively study the effect of occlusion in human activity recognition tasks; and (b) we aimed to assess the effect of the inclusion of occluded samples within the training process. As expected, occlusion is responsible for the loss of parts of motion, which in many cases are crucial for the recognition of an activity. Since to the best of our knowledge there do not exist any publicly available datasets, we used artificially-created occluded data and worked under the hypothesis that one or more body parts remain occluded during the whole duration of the activity. Of course this is often a harder case than those that are encountered in typical, real-life occlusion scenarios, wherein occlusion often does not affect a whole body part, but some of its joints instead. Additionally, in such scenarios, occlusion is not continuous. Some parts may remain occluded for some time intervals. Thus, it is our belief that the proposed approach constitutes a harder problem than typical occlusion cases.

To tackle occlusion, we followed a typical recognition approach. We first created 2D representations of human joint motion. Then we trained a Convolutional Neural Network both with and without artificially occluded samples. We evaluated our approach in typical human activity recognition scenarios, i.e., single-view, cross-view, and cross-subject, using two publicly available, large-scale datasets and upon considering eight occlusion cases involving one or two arms and/or legs. Prior to the experimental evaluation we expected that (a) the inclusion of occluded samples will boost the performance of recognition in the presence of occlusion; and (b) since most activities depend mainly on arms’ motion, recognition will be mostly affected in cases of arm occlusion. Both the aforementioned hypotheses have been experimentally proved. To our surprise, we also observed that the augmentation of the training set with occluded samples led to a boost of performance. We consider this as the most important finding of this work, since it may constitute a simple and effective approach for data augmentation in future work.

Future research efforts could focus on several other important aspects of the problem of occlusion. Firstly, we would like to investigate cases such as temporally partial occlusion. Moreover, we intend to investigate the use of other deep neural network architectures, such as generative adversarial networks (GANs) or approaches such as deep regression, for completing missing information. Finally, we plan to construct a dataset containing “real” occluded samples and also perform real-life experiments.

## Figures and Tables

**Table 1:** Results of the evaluation using the 11 activities of daily living of the PKU-MMD dataset. Setup cases are Cross-Subject (CS), Single-View (SV), and Cross-View (CV). Camera views are Left (L), Right (R), and Middle (M). In the baseline case (B), all body parts are included in both training and testing, while in the “simple” case of occlusion (S), the training set consists of full body parts and the test set consists of occluded body parts, and in the “augmented” case of occlusion (A), the training set consists of full and occluded body parts, while the test set consists of occluded body parts. Moreover, LA, RA, LL, and RL denote the occlusion of left arm, right arm, left leg, and right leg, respectively. F${}_{1}$ and Acc. denote F${}_{1}$ score and accuracy, respectively. In all cases, bold numbers indicate the best result between S and A cases.

Setup	Train	Test	Metric	B	LA		LA + LL		LL		LA + RA		LL + RL		RA		RA + RL		RL	
					S	A	S	A	S	A	S	A	S	A	S	A	S	A	S	A
**CS**	**LRM**	**LRM**	F${}_{1}$	0.68	0.56	**0.71**	0.53	**0.71**	0.62	**0.74**	0.18	**0.59**	0.47	**0.69**	0.53	**0.70**	0.49	**0.69**	0.66	**0.73**
			Acc.	0.70	0.59	**0.74**	0.55	**0.75**	0.64	**0.77**	0.28	**0.64**	0.52	**0.72**	0.55	**0.73**	0.50	**0.71**	0.67	**0.75**
**SV**	**L**	**L**	F${}_{1}$	0.68	0.51	**0.76**	0.49	**0.77**	0.70	**0.77**	0.18	**0.68**	0.58	**0.76**	0.40	**0.70**	0.37	**0.72**	0.66	**0.75**
			Acc.	0.69	0.54	**0.76**	0.54	**0.77**	0.71	**0.78**	0.29	**0.69**	0.60	**0.76**	0.42	**0.70**	0.40	**0.71**	0.66	**0.76**
	**M**	**M**	F${}_{1}$	0.77	0.66	**0.81**	0.62	**0.79**	0.74	**0.81**	0.27	**0.72**	0.63	**0.76**	0.63	**0.80**	0.57	**0.75**	0.74	**0.79**
			Acc.	0.78	0.67	**0.81**	0.64	**0.79**	0.75	**0.81**	0.37	**0.72**	0.66	**0.76**	0.65	**0.79**	0.59	**0.75**	0.74	**0.79**
	**R**	**R**	F${}_{1}$	0.75	0.54	**0.75**	0.49	**0.71**	0.71	**0.71**	0.16	**0.63**	0.64	**0.70**	0.59	**0.71**	0.59	**0.73**	0.72	**0.73**
			Acc.	0.76	0.57	**0.76**	0.53	**0.72**	0.72	**0.72**	0.29	**0.66**	0.65	**0.71**	0.62	**0.72**	0.60	**0.74**	0.72	**0.74**
**CV**	**L**	**M**	F${}_{1}$	0.71	0.59	**0.78**	0.58	**0.77**	0.69	**0.79**	0.22	**0.70**	0.60	**0.76**	0.61	**0.75**	0.60	**0.74**	0.70	**0.79**
			Acc0.	0.72	0.62	**0.78**	0.60	**0.78**	0.70	**0.79**	0.32	**0.70**	0.63	**0.77**	0.64	**0.75**	0.62	**0.74**	0.71	**0.79**
	**L**	**R**	F${}_{1}$	0.70	0.50	**0.64**	0.46	**0.63**	0.66	**0.67**	0.15	**0.65**	0.54	**0.67**	0.50	**0.68**	0.47	**0.69**	0.64	**0.69**
			Acc.	0.70	0.52	**0.65**	0.48	**0.64**	0.66	**0.68**	0.24	**0.66**	0.58	**0.67**	0.53	**0.69**	0.51	**0.69**	0.65	**0.70**
	**M**	**L**	F${}_{1}$	0.71	0.58	**0.77**	0.56	**0.77**	0.68	**0.76**	0.19	**0.67**	0.61	**0.71**	0.48	**0.71**	0.44	**0.69**	0.70	**0.74**
			Acc.	0.72	0.62	**0.78**	0.60	**0.77**	0.69	**0.76**	0.32	**0.69**	0.63	**0.72**	0.50	**0.71**	0.47	**0.68**	0.70	**0.74**
	**M**	**R**	F${}_{1}$	0.55	0.35	**0.66**	0.34	**0.63**	0.52	**0.69**	0.15	**0.51**	0.40	**0.64**	0.42	**0.70**	0.38	**0.70**	0.50	**0.70**
			Acc.	0.56	0.41	**0.67**	0.39	**0.64**	0.53	**0.70**	0.26	**0.56**	0.43	**0.66**	0.44	**0.71**	0.40	**0.72**	0.51	**0.71**
	**R**	**L**	F${}_{1}$	0.60	0.53	**0.62**	0.48	**0.60**	0.55	**0.59**	0.18	**0.48**	0.44	**0.56**	0.49	**0.57**	0.48	**0.55**	0.58	**0.57**
			Acc.	0.59	0.54	**0.64**	0.49	**0.62**	0.54	**0.59**	0.29	**0.53**	0.47	**0.56**	0.50	**0.57**	0.49	**0.55**	0.58	**0.57**
	**R**	**M**	F${}_{1}$	0.73	0.60	**0.70**	0.52	**0.69**	0.65	**0.74**	0.25	**0.61**	0.57	**0.72**	0.60	**0.71**	0.57	**0.70**	0.72	**0.74**
			Acc.	0.73	0.61	**0.71**	0.54	**0.70**	0.66	**0.74**	0.34	**0.63**	0.60	**0.72**	0.60	**0.72**	0.57	**0.71**	0.72	**0.74**
	**LR**	**M**	F${}_{1}$	0.87	0.70	**0.88**	0.67	**0.87**	0.84	**0.90**	0.23	**0.80**	0.68	**0.88**	0.60	**0.88**	0.56	**0.88**	0.83	**0.89**
			Acc.	0.87	0.71	**0.88**	0.68	**0.86**	0.85	**0.90**	0.36	**0.79**	0.71	**0.88**	0.64	**0.88**	0.59	**0.88**	0.83	**0.89**
	**LM**	**R**	F${}_{1}$	0.79	**0.67**	0.65	**0.65**	0.62	**0.76**	0.68	0.21	**0.52**	0.64	**0.64**	0.56	**0.66**	0.53	**0.67**	**0.77**	0.70
			Acc.	0.79	**0.68**	0.66	**0.66**	0.63	**0.76**	0.69	0.34	**0.54**	**0.66**	0.65	0.59	**0.66**	0.55	0.67	**0.77**	0.70
	**RM**	**L**	F${}_{1}$	0.74	0.68	**0.79**	0.69	**0.79**	0.74	**0.78**	0.22	**0.68**	0.62	**0.71**	0.55	**0.73**	0.54	**0.71**	0.72	**0.76**
			Acc.	0.75	0.69	**0.80**	0.70	**0.80**	0.74	**0.77**	0.34	**0.69**	0.63	**0.71**	0.57	**0.73**	0.56	**0.70**	0.72	**0.76**

**Table 2:** Results of the evaluation using the 12 medical conditions of the NTU-RGB+d dataset. Setup cases are Cross-Subject (CS), Single-View (SV), and Cross-View (CV). Camera views are Left (L), Right (R), and Middle (M). In the baseline case (B), all body parts are included in both training and testing, while in the “simple” case of occlusion (S) the training set consists of full body parts and the test set consists of occluded body parts, and in the “augmented” case of occlusion (A), the training set consists of full and occluded body parts, while the test set consists of occluded body parts. LA, RA, LL, and RL denote the occlusion of left arm, right arm, left leg, and right leg, respectively. F${}_{1}$ and Acc. denote F${}_{1}$ score and accuracy, respectively. In all cases, bold numbers indicate the best result between S and A cases.

Setup	Train	Test	Metric	B	LA		LA + LL		LL		LA + RA		LL + RL		RA		RA + RL		RL	
					S	A	S	A	S	A	S	A	S	A	S	A	S	A	S	A
**CS**	**LRM**	**LRM**	F${}_{1}$	0.61	0.30	**0.55**	0.32	**0.54**	0.59	**0.64**	0.18	**0.44**	0.51	**0.64**	0.43	**0.61**	0.43	**0.62**	0.58	**0.66**
			Acc.	0.61	0.32	**0.54**	0.33	**0.54**	0.62	**0.64**	0.22	**0.42**	0.58	**0.65**	0.44	**0.62**	0.44	**0.62**	0.58	**0.66**
**SV**	**L**	**L**	F${}_{1}$	0.60	0.35	**0.54**	0.37	**0.54**	0.58	**0.64**	0.17	**0.41**	0.46	**0.63**	0.45	**0.61**	0.43	**0.62**	0.57	**0.66**
			Acc.	0.61	0.38	**0.55**	0.39	**0.54**	0.59	**0.64**	0.24	**0.44**	0.48	**0.64**	0.47	**0.62**	0.45	**0.62**	0.57	**0.66**
	**M**	**M**	F${}_{1}$	0.49	0.28	**0.50**	0.30	**0.49**	0.49	**0.59**	0.12	**0.42**	0.36	**0.61**	0.38	**0.59**	0.37	**0.60**	0.46	**0.61**
			Acc.	0.50	0.30	**0.50**	0.32	**0.49**	0.49	**0.60**	0.19	**0.43**	0.38	**0.61**	0.41	**0.59**	0.38	**0.60**	0.46	**0.62**
	**R**	**R**	F${}_{1}$	0.53	0.29	**0.48**	0.30	**0.48**	0.51	**0.59**	0.11	**0.37**	0.44	**0.62**	0.39	**0.54**	0.37	**0.55**	0.52	**0.61**
			Acc.	0.54	0.31	**0.48**	0.32	**0.48**	0.52	**0.59**	0.19	**0.37**	0.46	**0.62**	0.42	**0.54**	0.40	**0.55**	0.52	**0.61**
**CV**	**L**	**M**	F${}_{1}$	0.50	0.25	**0.50**	0.26	**0.51**	0.43	**0.63**	0.14	**0.38**	0.36	**0.63**	0.39	**0.62**	0.36	**0.64**	0.47	**0.64**
			Acc.	0.50	0.28	**0.50**	0.27	**0.51**	0.43	**0.63**	0.19	**0.40**	0.39	**0.63**	0.39	**0.62**	0.37	**0.64**	0.46	**0.64**
	**L**	**R**	F${}_{1}$	0.50	0.33	**0.50**	0.34	**0.51**	0.49	**0.58**	0.18	**0.37**	0.42	**0.58**	0.38	**0.53**	0.37	**0.54**	0.48	**0.58**
			Acc.	0.50	0.35	**0.50**	0.35	**0.51**	0.49	**0.58**	0.22	**0.37**	0.43	**0.58**	0.39	**0.53**	0.38	**0.55**	0.47	**0.59**
	**M**	**L**	F${}_{1}$	0.59	0.31	**0.51**	0.33	**0.51**	0.55	**0.64**	0.13	**0.36**	0.47	**0.63**	0.45	**0.58**	0.44	**0.58**	0.55	**0.64**
			Acc.	0.59	0.33	**0.51**	0.34	**0.51**	0.55	**0.64**	0.19	**0.39**	0.49	**0.63**	0.47	**0.59**	0.44	**0.58**	0.55	**0.65**
	**M**	**R**	F${}_{1}$	0.61	0.37	**0.42**	0.36	**0.45**	**0.57**	0.53	0.12	**0.30**	0.49	**0.54**	0.42	**0.49**	0.40	**0.50**	**0.56**	0.53
			Acc.	0.61	0.38	**0.43**	0.38	**0.45**	**0.57**	0.54	0.18	**0.31**	0.51	**0.54**	0.44	**0.49**	0.41	**0.50**	**0.56**	0.54
	**R**	**L**	F${}_{1}$	0.59	0.28	**0.51**	0.31	**0.51**	0.57	**0.61**	0.11	**0.39**	0.49	**0.61**	0.43	**0.56**	0.42	**0.58**	0.55	**0.62**
			Acc.	0.59	0.31	**0.52**	0.32	**0.52**	0.57	**0.62**	0.18	**0.41**	0.50	**0.61**	0.44	**0.56**	0.43	**0.58**	0.55	**0.63**
	**R**	**M**	F${}_{1}$	0.55	0.34	**0.39**	0.34	**0.39**	**0.52**	0.47	0.17	**0.29**	0.46	**0.47**	0.43	**0.46**	0.41	**0.49**	0.49	**0.49**
			Acc.	0.55	0.35	**0.40**	0.34	**0.39**	**0.52**	0.47	0.21	**0.31**	**0.49**	0.47	0.42	**0.46**	0.42	**0.48**	**0.51**	0.49
	**LR**	**M**	F${}_{1}$	0.57	0.32	**0.51**	0.31	**0.52**	0.53	**0.61**	0.17	**0.44**	0.44	**0.64**	0.44	**0.62**	0.44	**0.65**	0.55	**0.64**
			Acc.	0.58	0.33	**0.52**	0.31	**0.52**	0.53	**0.62**	0.22	**0.45**	0.46	**0.64**	0.46	**0.63**	0.46	**0.65**	0.56	**0.65**
	**LM**	**R**	F${}_{1}$	0.60	0.28	**0.51**	0.29	**0.52**	0.54	**0.61**	0.14	**0.37**	0.47	**0.61**	0.44	**0.55**	0.43	**0.56**	0.56	**0.60**
			Acc.	0.60	0.31	**0.52**	0.30	**0.53**	0.54	**0.61**	0.18	**0.38**	0.50	**0.61**	0.45	**0.55**	0.43	**0.56**	0.56	**0.61**
	**RM**	**L**	F${}_{1}$	0.65	0.38	**0.58**	0.39	**0.59**	0.62	**0.72**	0.14	**0.44**	0.52	**0.72**	0.48	**0.68**	0.46	**0.69**	0.62	**0.72**
			Acc.	0.65	0.39	**0.58**	0.39	**0.59**	0.62	**0.73**	0.21	**0.45**	0.55	**0.72**	0.50	**0.69**	0.48	**0.69**	0.62	**0.73**

**Table 3:** Results of the evaluation using the PKU-MMD dataset. Setup cases are Cross-Subject (CS), Single-View (SV), and Cross-View (CV). Camera views are Left (L), Right (R), and Middle (M). In the baseline case (B), all body parts are included in both training and testing, while in the “simple” case of occlusion (S) the training set consists of full body parts and the test set consists of occluded body parts, and in the “augmented” case of occlusion (A), the training set consists of full and occluded body parts, while the test set consists of occluded body parts. LA, RA, LL, and RL denote the occlusion of left arm, right arm, left leg, and right leg, respectively. F${}_{1}$ and Acc. denote F${}_{1}$ score and accuracy, respectively. In all cases, bold numbers indicate the best result between S and A cases.

Setup	Train	Test	Metric	B	LA		LA + LL		LL		LA + RA		LL + RL		RA		RA + RL		RL	
					S	A	S	A	S	A	S	A	S	A	S	A	S	A	S	A
**CS**	**LRM**	**LRM**	F${}_{1}$	0.69	0.29	**0.51**	0.25	**0.48**	0.65	**0.64**	0.10	**0.34**	0.60	**0.62**	0.39	**0.59**	0.36	**0.58**	0.65	**0.65**
			Acc.	0.70	0.33	**0.53**	0.29	**0.51**	0.66	**0.66**	0.17	**0.37**	0.62	**0.65**	0.45	**0.61**	0.42	**0.60**	0.66	**0.67**
**SV**	**L**	**L**	F${}_{1}$	0.64	0.29	**0.52**	0.27	**49**	0.58	**0.65**	0.09	**0.37**	0.50	**0.63**	0.30	**0.60**	0.28	**0.60**	0.61	**0.65**
			Acc.	0.65	0.33	**0.53**	0.30	**0.50**	0.59	**0.66**	0.16	**0.39**	0.52	**0.64**	0.36	**0.60**	0.35	**0.60**	0.61	**0.66**
	**M**	**M**	F${}_{1}$	0.63	0.26	**0.54**	0.21	**0.51**	0.59	**0.67**	0.09	**38**	0.50	**0.67**	0.36	**0.65**	0.33	**0.65**	0.62	**0.68**
			Acc.	0.64	0.29	**0.55**	0.26	**0.52**	0.60	**0.68**	0.15	**0.41**	0.53	**0.68**	0.41	**0.65**	0.39	**0.65**	0.62	**0.69**
	**R**	**R**	F${}_{1}$	0.63	0.29	**0.53**	0.26	**0.53**	0.60	**0.65**	0.09	**0.37**	0.56	**0.62**	0.38	**0.64**	0.33	**0.63**	0.61	**0.65**
			Acc.	0.63	0.32	**0.54**	0.30	**0.54**	0.60	**0.65**	0.15	**0.39**	0.57	**0.63**	0.42	**0.64**	0.38	**0.63**	0.62	**0.65**
**CV**	**L**	**M**	F${}_{1}$	0.61	0.23	**0.50**	0.22	**0.47**	0.55	**0.64**	0.10	**0.34**	0.49	**0.63**	0.34	**0.60**	0.32	**0.60**	0.59	**0.65**
			Acc.	0.62	0.27	**0.51**	0.26	**0.48**	0.56	**0.65**	0.15	**0.37**	0.53	**0.64**	0.39	**0.61**	0.37	**0.61**	0.59	**0.66**
	**L**	**R**	F${}_{1}$	0.58	0.23	**0.34**	0.21	**0.33**	**0.53**	0.44	0.08	**0.27**	**0.45**	0.44	0.36	**0.42**	0.32	**0.42**	**0.56**	0.46
			Acc.	0.59	0.28	**0.37**	0.25	**0.35**	**0.54**	0.46	0.14	**0.30**	**0.49**	0.46	0.40	**0.44**	0.36	**0.45**	**0.57**	0.48
	**M**	**L**	F${}_{1}$	0.61	0.28	**0.53**	0.26	**0.52**	0.54	**0.63**	0.08	**0.34**	0.49	**0.62**	0.34	**0.58**	0.33	**0.58**	0.58	**0.64**
			Acc.	0.62	0.31	**0.54**	0.28	**0.53**	0.56	**0.64**	0.14	**0.37**	0.51	**0.63**	0.39	**0.59**	0.38	**0.58**	0.60	**0.65**
	**M**	**R**	F${}_{1}$	0.50	0.20	**0.47**	0.18	**0.46**	0.45	**0.62**	0.09	**0.34**	0.39	**0.62**	0.29	**0.61**	0.24	**0.61**	0.47	**0.63**
			Acc.	0.51	0.24	**0.49**	0.22	**0.47**	0.47	**0.63**	0.14	**0.36**	0.41	**0.62**	0.32	**0.61**	0.27	**0.61**	0.47	**0.63**
	**R**	**L**	F${}_{1}$	0.47	0.18	**0.38**	0.16	**0.37**	0.41	**0.47**	0.08	**0.25**	0.37	**0.44**	0.26	**0.44**	0.26	**0.43**	0.44	**0.48**
			Acc.	0.48	0.22	**0.40**	0.19	**0.39**	0.43	**0.49**	0.16	**0.28**	0.40	**0.47**	0.32	**0.46**	0.33	**0.45**	0.46	**0.50**
	**R**	**M**	F${}_{1}$	0.60	0.25	**0.48**	0.22	**0.47**	0.54	**0.62**	0.08	**0.33**	0.46	**0.61**	0.38	**0.58**	0.33	**0.57**	0.57	**0.64**
			Acc.	0.61	0.30	**0.50**	0.24	**0.48**	0.54	**0.63**	0.16	**0.35**	0.48	**0.62**	0.42	**0.58**	0.37	**0.58**	0.57	**0.64**
	**LR**	**M**	F${}_{1}$	0.70	0.31	**0.58**	0.26	**0.56**	0.65	**0.74**	0.09	**0.40**	0.60	**0.73**	0.42	**0.69**	0.38	**0.69**	0.68	**0.75**
			Acc.	0.70	0.34	**0.58**	0.30	**0.56**	0.67	**0.74**	0.15	**0.41**	0.63	**0.73**	0.46	**0.69**	0.43	**0.68**	0.68	**0.75**
	**LM**	**R**	F${}_{1}$	0.65	0.26	**0.50**	0.25	**0.49**	0.61	**0.63**	0.11	**0.36**	0.56	**0.62**	0.36	**0.61**	0.35	**0.62**	0.60	**0.64**
			Acc.	0.66	0.30	**0.52**	0.29	**0.50**	0.62	**0.64**	0.17	**0.39**	0.58	**64**	0.42	**0.62**	0.41	**0.62**	0.61	**0.65**
	**RM**	**L**	F${}_{1}$	0.65	0.27	**0.50**	0.26	**0.49**	0.60	**0.62**	0.09	**0.34**	0.55	**0.60**	0.36	**0.56**	0.35	**0.55**	0.61	**0.62**
			Acc.	0.65	0.30	**0.51**	0.30	**0.49**	0.61	**0.63**	0.15	**0.36**	0.56	**0.60**	0.40	**0.57**	0.40	**0.56**	0.62	**0.63**

**Table 4:** Results of the evaluation using the NTU-RGB+D dataset. Setup cases are Cross-Subject (CS), Single-View (SV), and Cross-View (CV). Camera views are Left (L), Right (R), and Middle (M). In the baseline case (B), all body parts are included in both training and testing, while in the “simple” case of occlusion (S) the training set consists of full body parts and the test set consists of occluded body parts, and in the “augmented” case of occlusion (A), the training set consists of full and occluded body parts, while the test set consists of occluded body parts. LA, RA, LL, and RL denote the occlusion of left arm, right arm, left leg, and right leg, respectively. F${}_{1}$ and Acc. denote F${}_{1}$ score and accuracy, respectively. In all cases, bold numbers indicate the best result between S and A cases.

Setup	Train	Test	Metric	B	LA		LA + LL		LL		LA + RA		LL + RL		RA		RA + RL		RL	
					S	A	S	A	S	A	S	A	S	A	S	A	S	A	S	A
**CS**	**LRM**	**LRM**	F${}_{1}$	0.49	0.25	**0.37**	0.22	**0.36**	**0.46**	0.45	0.09	**0.25**	0.39	**0.45**	0.31	**0.44**	0.29	**0.44**	0.47	**0.47**
			Acc.	0.49	0.26	**0.38**	0.23	**0.37**	**0.47**	0.47	0.12	**0.27**	0.41	**0.47**	0.32	**0.45**	0.31	**0.45**	0.47	**0.48**
**SV**	**L**	**L**	F${}_{1}$	0.60	0.25	**0.39**	0.20	**0.38**	**0.50**	0.49	0.08	**0.28**	0.37	**0.50**	0.34	**0.46**	0.29	**0.48**	**0.53**	0.51
			Acc.	0.60	0.26	**0.40**	0.21	**0.39**	**0.51**	0.50	0.11	**0.29**	0.41	**0.50**	0.36	**0.46**	0.32	**0.48**	**0.55**	0.51
	**M**	**M**	F${}_{1}$	0.57	0.27	**0.36**	0.24	**0.35**	**0.50**	0.46	0.09	**0.24**	0.42	**0.46**	0.36	**0.44**	0.31	**0.43**	**0.51**	0.48
			Acc.	0.58	0.28	**0.37**	0.25	**0.36**	**0.51**	0.47	0.11	**0.26**	0.45	**0.47**	0.38	**0.44**	0.32	**0.44**	**0.52**	0.49
	**R**	**R**	F${}_{1}$	0.58	0.27	**0.35**	0.22	**0.35**	**0.51**	0.44	0.08	**0.25**	0.40	**0.46**	0.32	**0.38**	0.27	**0.40**	**0.52**	0.47
			Acc.	0.59	0.29	**0.36**	0.24	**0.35**	**0.52**	0.45	0.10	**0.27**	0.43	**0.46**	0.34	**0.38**	0.30	**0.40**	**0.53**	0.47
**CV**	**L**	**M**	F${}_{1}$	0.38	0.14	**0.35**	0.11	**0.34**	0.35	**0.45**	0.04	**0.24**	0.28	**0.46**	0.23	**0.43**	0.20	**0.44**	0.35	**0.47**
			Acc.	0.37	0.16	**0.36**	0.13	**0.35**	0.35	**0.46**	0.06	**0.26**	0.30	**0.46**	0.23	**0.43**	0.20	**0.44**	0.34	**0.47**
	**L**	**R**	F${}_{1}$	0.41	0.24	**0.35**	0.20	**0.35**	0.37	**0.43**	0.09	**0.23**	0.31	**0.42**	0.26	**0.39**	0.25	**0.39**	0.39	**0.44**
			Acc.	0.41	0.24	**0.35**	0.20	**0.35**	0.37	**0.44**	0.12	**0.23**	0.33	**0.43**	0.29	**0.39**	0.28	**0.39**	0.40	**0.44**
	**M**	**L**	F${}_{1}$	0.46	0.23	**0.39**	0.20	**0.38**	0.44	**0.49**	0.08	**0.25**	0.35	**0.49**	0.31	**0.45**	0.29	**0.45**	0.45	**0.50**
			Acc.	0.46	0.24	**0.39**	0.21	**0.38**	0.44	**0.50**	0.10	**0.26**	0.37	**0.49**	0.32	**0.45**	0.32	**0.45**	0.45	**0.51**
	**M**	**R**	F${}_{1}$	0.46	0.26	**0.33**	0.25	**0.32**	**0.43**	0.40	0.09	**0.22**	0.37	**0.39**	0.30	**0.37**	0.28	**0.37**	0.45	**0.41**
			Acc.	0.46	0.28	**0.33**	0.26	**0.32**	**0.44**	0.40	0.11	**0.23**	0.39	**0.40**	0.32	**0.37**	0.30	**0.37**	0.46	**0.41**
	**R**	**L**	F${}_{1}$	0.44	0.23	**0.33**	0.22	**0.33**	0.41	**0.44**	0.08	**0.21**	0.35	**0.43**	0.27	**0.39**	0.25	**0.39**	0.42	**0.45**
			Acc.	0.43	0.24	**0.34**	0.23	**0.33**	0.41	**0.44**	0.10	**0.22**	0.37	**0.44**	0.28	**0.38**	0.27	**0.39**	0.42	**0.46**
	**R**	**M**	F${}_{1}$	0.50	0.23	**0.31**	0.20	**0.30**	**0.47**	0.38	0.09	**0.23**	**0.42**	0.39	**0.32**	0.38	0.28	**0.39**	**0.47**	0.40
			Acc.	0.50	0.24	**0.32**	0.21	**0.31**	**0.48**	0.39	0.10	**0.25**	**0.43**	0.39	**0.33**	0.39	0.30	**0.39**	**0.47**	0.41
	**LR**	**M**	F${}_{1}$	0.42	0.22	**0.37**	0.21	**0.36**	0.40	**0.46**	0.07	**0.26**	0.35	**0.46**	0.27	**0.44**	0.26	**0.44**	0.41	**0.48**
			Acc.	0.43	0.23	**0.38**	0.22	**0.37**	0.42	**0.47**	0.10	**0.28**	0.37	**0.47**	0.30	**0.44**	0.28	**0.45**	0.43	**0.49**
	**LM**	**R**	F${}_{1}$	0.46	0.24	**0.34**	0.22	**0.33**	**0.43**	0.42	0.09	**0.22**	0.38	**0.41**	0.27	**0.37**	0.26	**0.37**	**0.44**	0.42
			Acc.	0.45	0.25	**0.35**	0.23	**0.34**	**0.43**	0.43	0.12	**0.24**	0.40	**0.42**	0.28	**0.38**	0.27	**0.38**	**0.44**	0.43
	**RM**	**L**	F${}_{1}$	0.52	0.29	**0.37**	0.24	**0.36**	0.48	**0.48**	0.07	**0.25**	0.40	**0.48**	0.32	**0.45**	0.28	**0.46**	0.48	**0.51**
			Acc.	0.52	0.29	**0.38**	0.25	**0.36**	0.49	**0.49**	0.10	**0.26**	0.43	**0.48**	0.34	**0.45**	0.31	**0.46**	0.50	**0.51**

## Data Availability

The PKU-MMD dataset is available at https://www.icst.pku.edu.cn/struct/Projects/PKUMMD.html (accessed on 6 July 2022). The NTU-RGB+D dataset is available at https://rose1.ntu.edu.sg/dataset/actionRecognition/ (accessed on 6 July 2022).

## Abbreviations

The following abbreviations are used in this manuscript:

ADL	Activity of Daily Living
CCTV	Closed Circuit Television
CNN	Convolutional Neural Network
CS	Cross-Subject
CV	Cross-View
GPU	Graphics Processing Unit
HAR	Human Activity Recognition
LA	Left Arm
LL	Left Leg
LSTM	Long-Short Term Memory
RA	Right Arm
RL	Right Leg
RFID	Radio Frequency Identification
SV	Single-View
